# Supplementary material for: Carbonate production of Micronesian reefs suppressed by thermal anomalies and Acanthaster as sea-level rises
Source: PLoS One. 2019 Nov 15;14(11):e0224887. doi: 10.1371/journal.pone.0224887 (PMC6857905; doi:10.1371/journal.pone.0224887)
Supplement: S1 File — These figures include (A) a location map, (B) limit on the number of Acanthaster solaris sea stars, as a proportion of live coral cover (LCC) that a 100 m2 shallow-water coral-reef habitat, (C and D) erosional kriged maps for both islands, and (E and F) erosional maps for parrotfishes for both islands, and (G) net vertical accretion split by strata compared to the rates of sea-level rise under representative concentration pathways 2.6, 4.5, 6.0, and 8.5 W m-2. (PDF) [file pone.0224887.s001.pdf]

S1 File.

Supplementary document to: Carbonate production of Micronesian reefs suppressed by thermal anomalies and *Acanthaster* as sea-level rises

Robert van Woesik and Christopher William Cacciapaglia

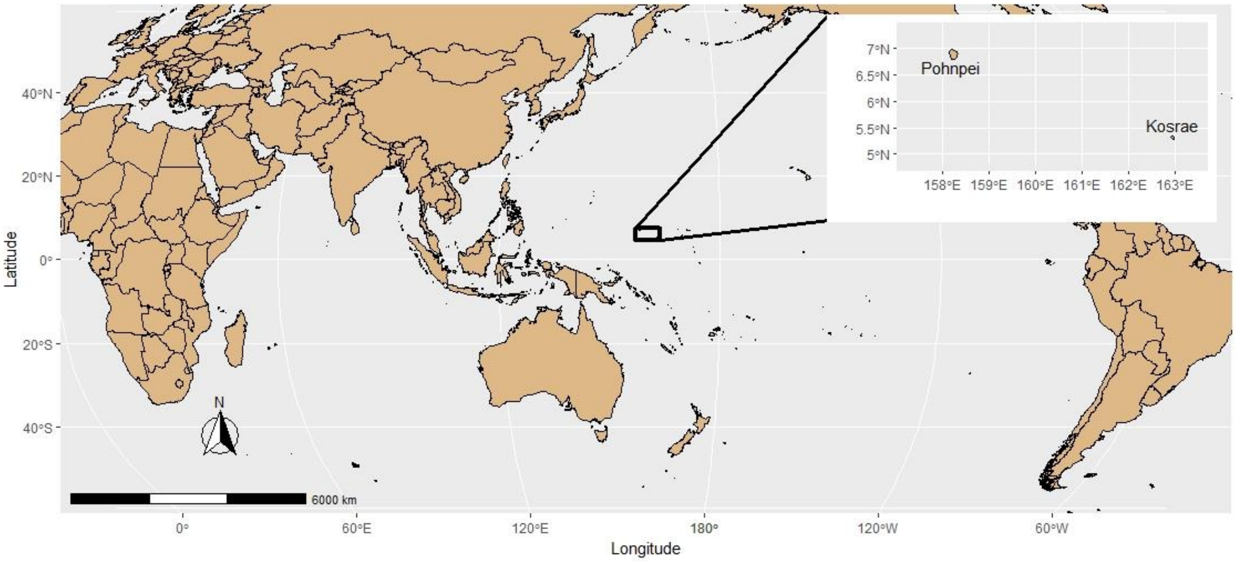

Figure A. Map of study sites.

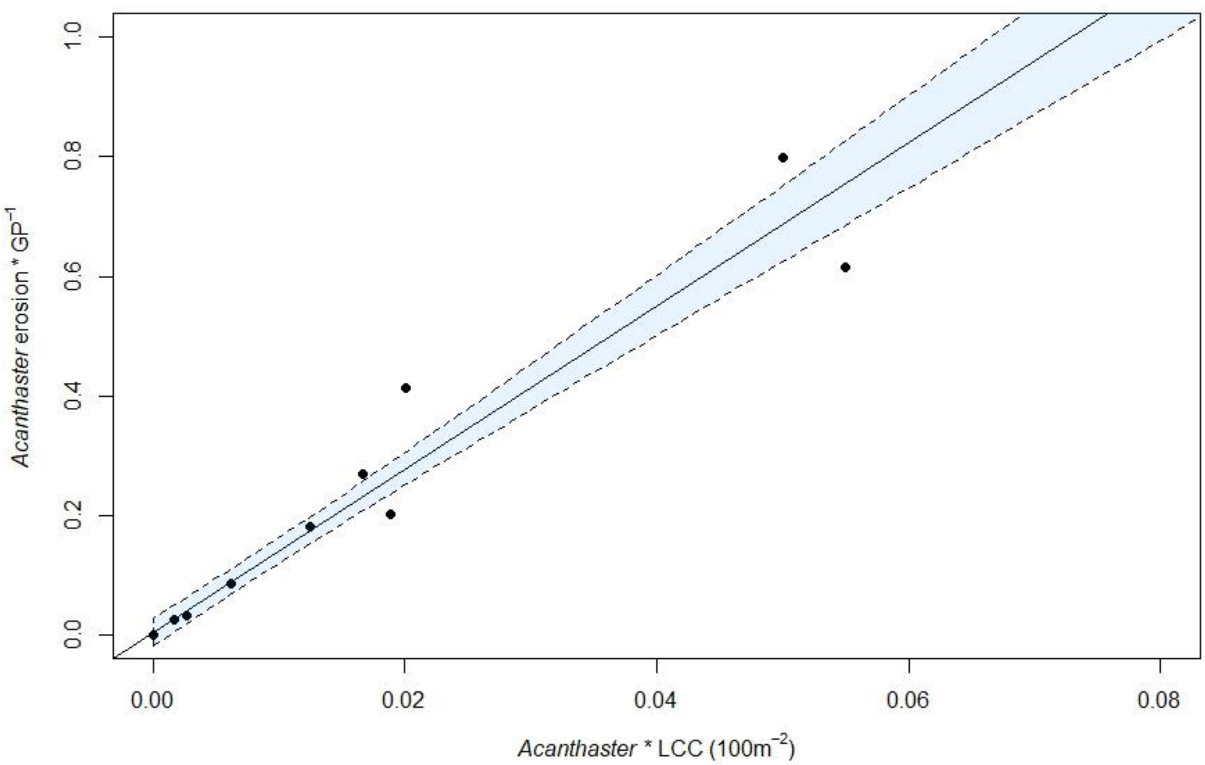

Figure B.

Limit on the number of *Acanthaster solaris* sea stars, as a proportion of live coral cover (LCC) that a 100 m^2 shallow-water coral-reef habitat can endure before erosional forces overtake gross production (GP) (equal to 1 on the y axis). The number of *Acanthaster solaris* where $y = 1$ (reducing gross production to 0) is at (0.073) 7.3% of LCC for a 100 m^2 area. These data were collected at a total of 48 sites in both Kosrae and Pohnpei inner reefs and outer reefs, and Pohnpei patch reefs, Federated States of Micronesia, 2018. The dots represent the data and the dashes represent the 95% confidence intervals.

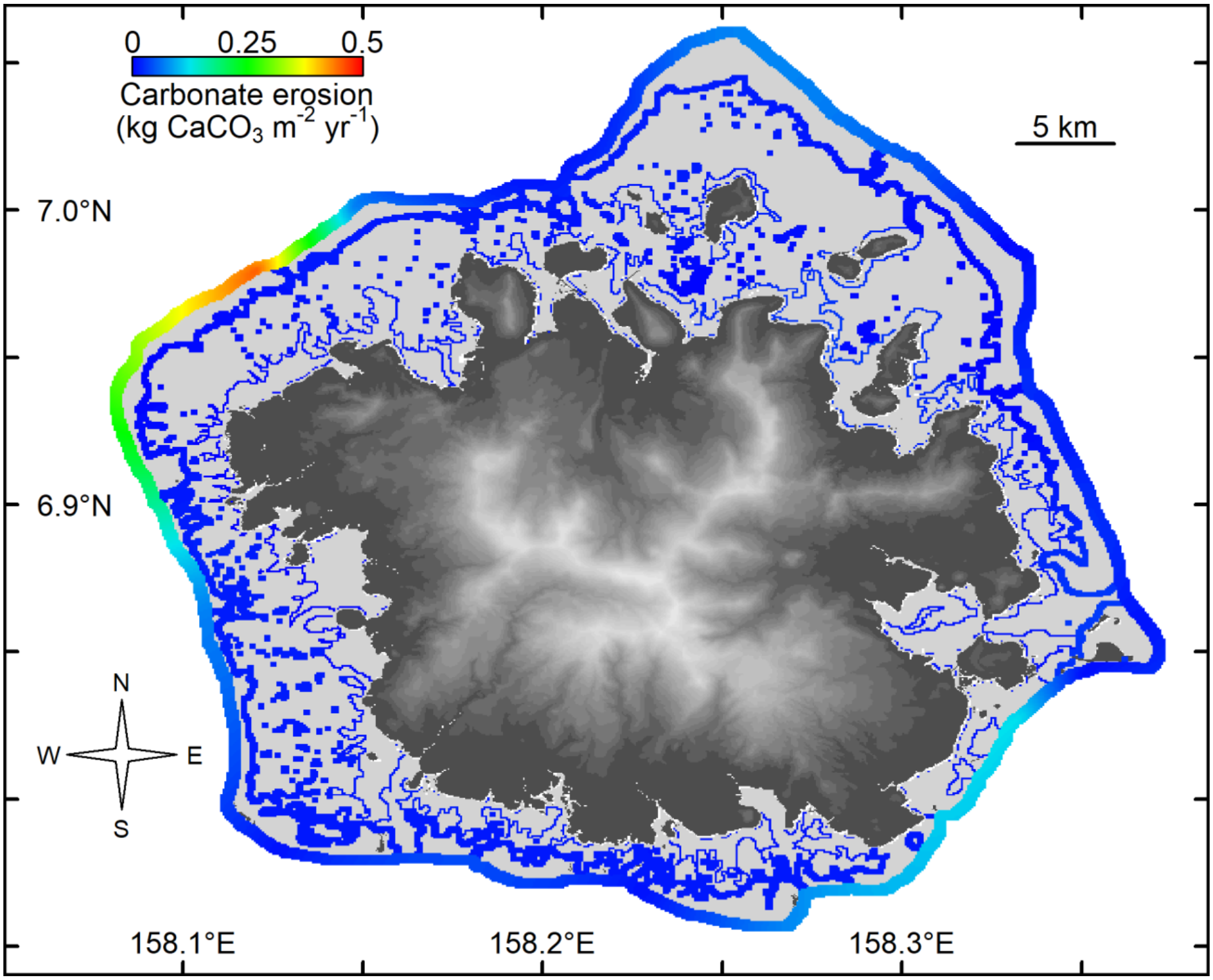

Figure C. Erosional estimates (kg $CaCO_3$ m^{-2} yr^{-1}) using spatial kriging for shallow-water coral-reef habitats (including inner, outer, and patch reefs) in Pohnpei, Federated States of Micronesia, 2018. Erosion includes parrotfish and urchin erosional forces combined (which does not include *Acanthaster solaris* erosion).

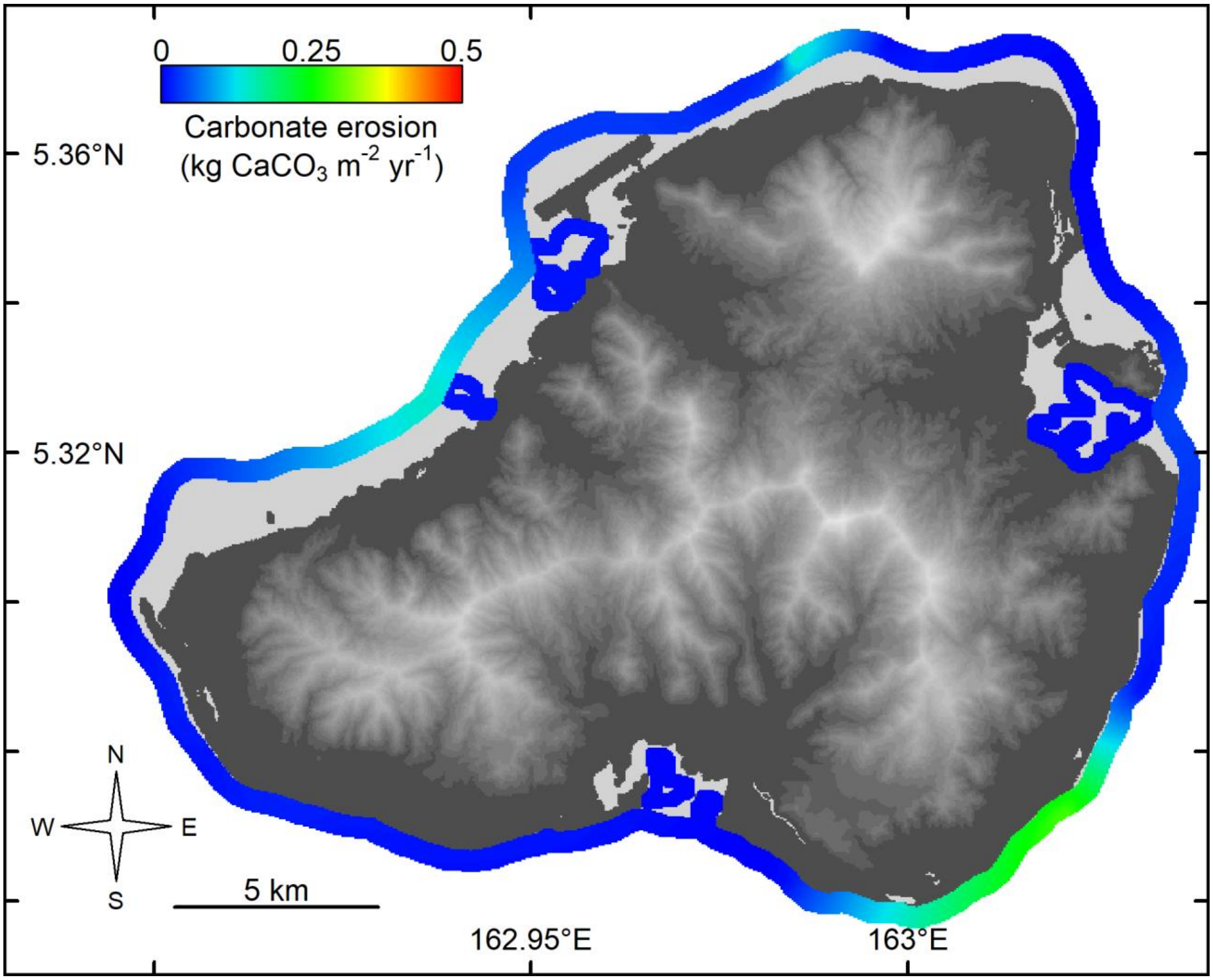

Figure D. Erosional estimates (kg $CaCO_3$ m^{-2} yr^{-1}) using spatial kriging for shallow-water coral-reef habitats (including inner and outer reefs) in Kosrae, Federated States of Micronesia, 2018. Erosion includes parrotfish and urchin erosional forces combined (which does not include *Acanthaster solaris* erosion).

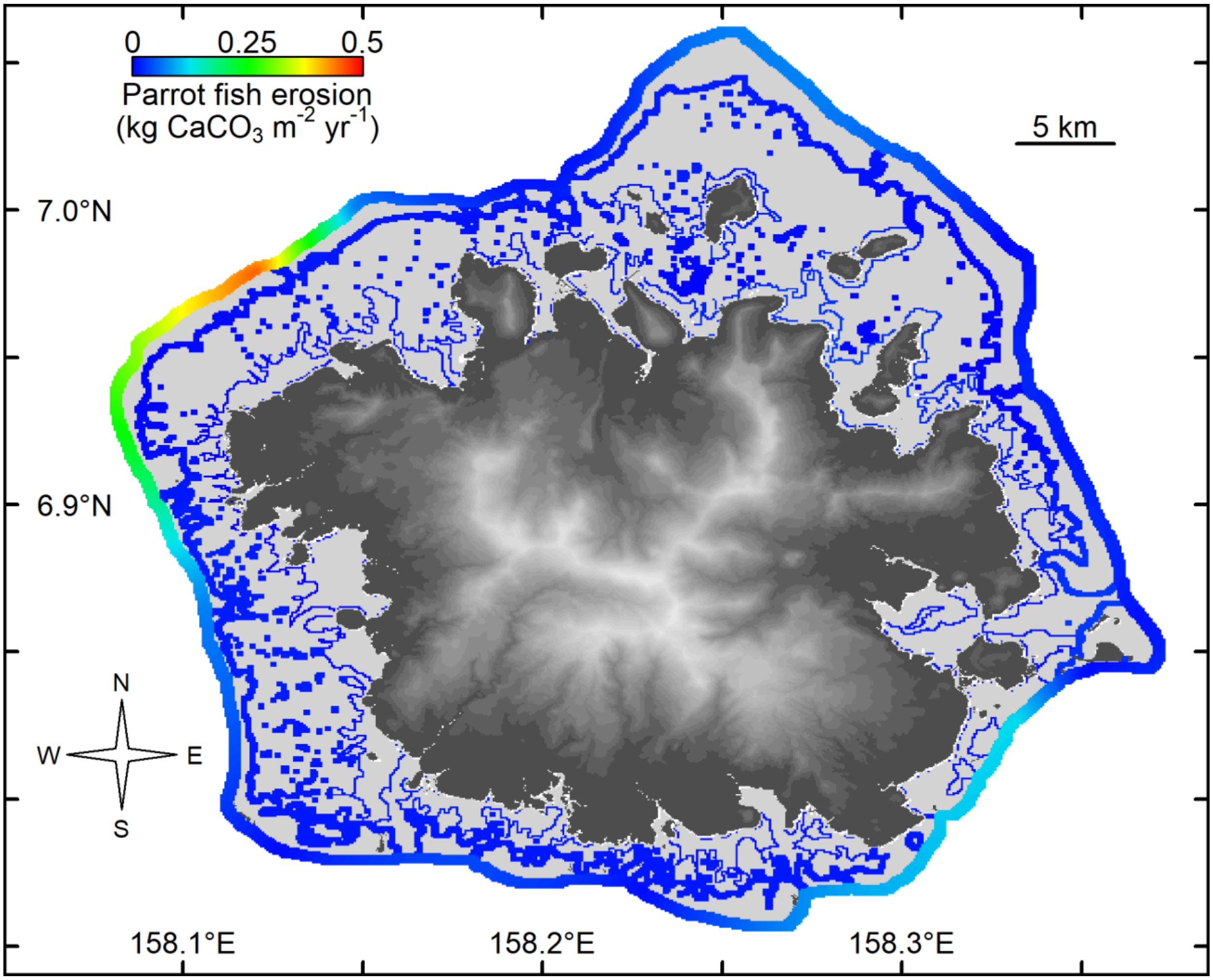

Figure E. Erosional estimates for parrotfishes (kg $CaCO_3$ m^{-2} yr^{-1}) using spatial kriging for shallow-water coral-reef habitats (including inner, outer, and patch reefs) in Pohnpei, Federated States of Micronesia, 2018.

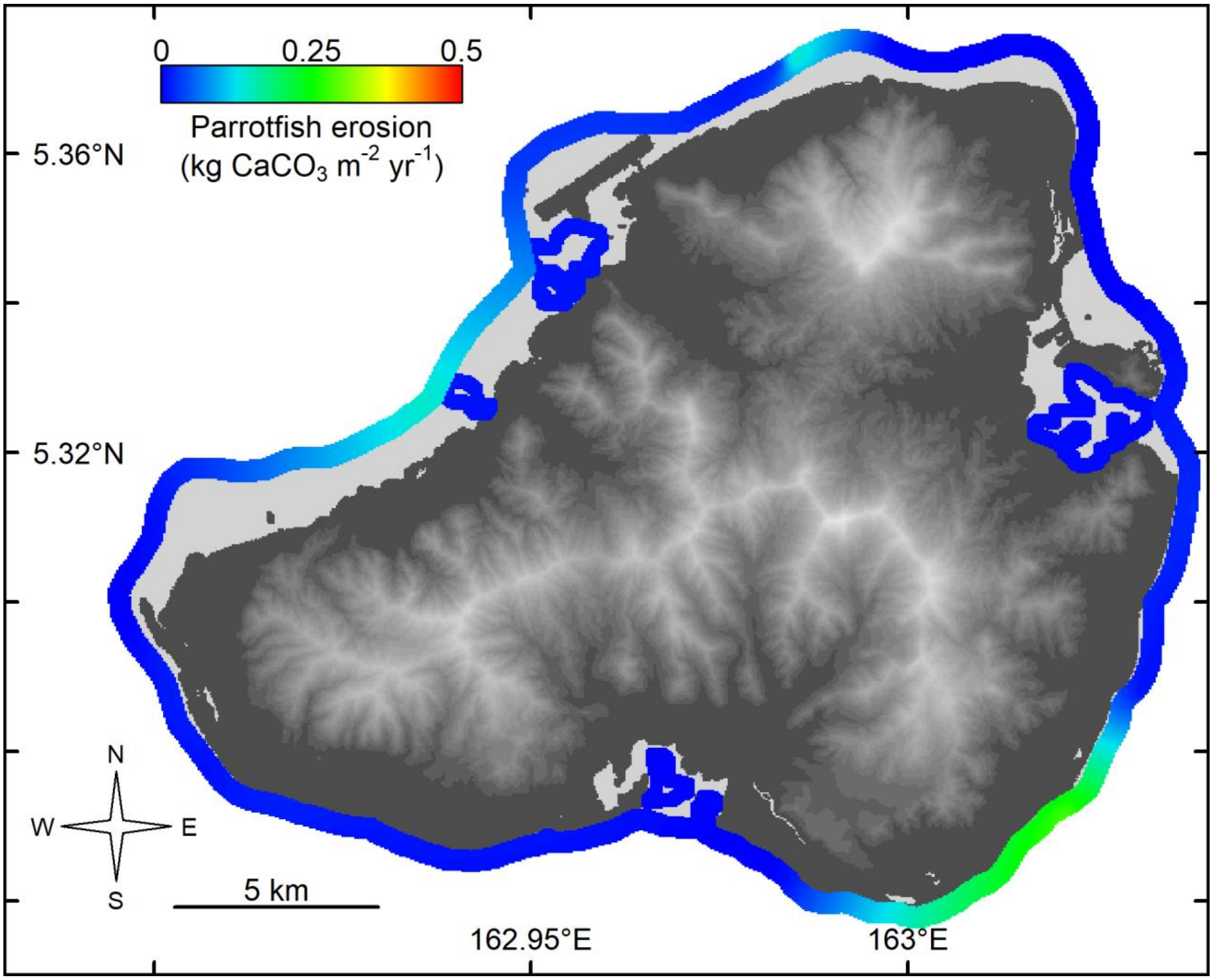

Figure F. Erosional estimates for parrotfishes (kg $CaCO_3$ m^{-2} yr^{-1}) using spatial kriging for shallow-water coral-reef habitats (including inner and outer reefs) in Kosrae, Federated States of Micronesia, 2018.

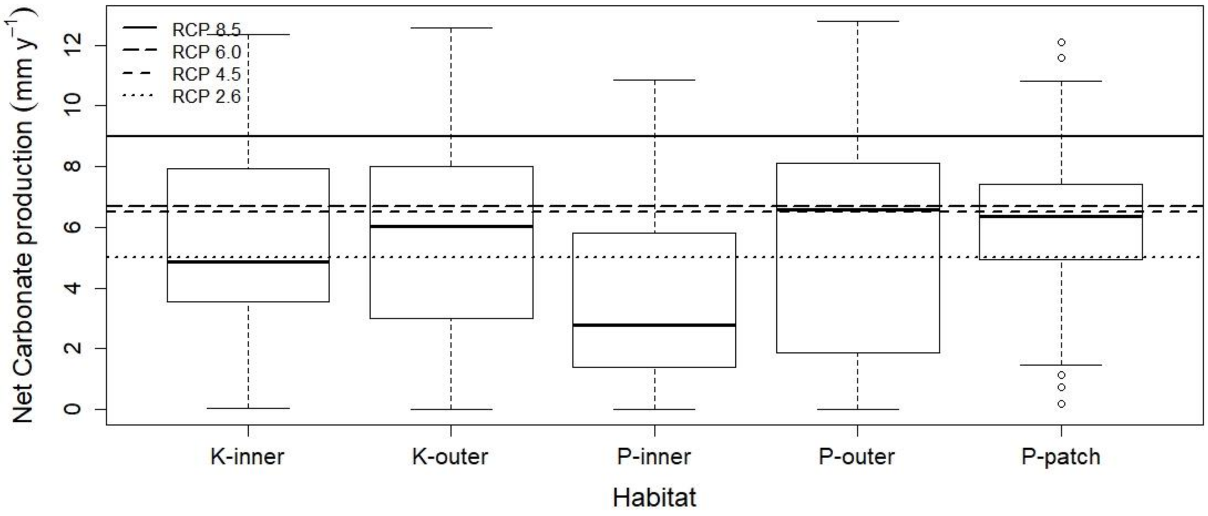

Figure G. Net vertical accretion split by strata compared to the rates of sea-level rise under representative concentration pathways 2.6, 4.5, 6.0, and 8.5 W m^{-2} as horizontal lines. Box and whisker plots indicate the median, as the thick horizontal line, the box surrounding the medians are the first and third quartiles, the whiskers identify the range of the data, and the circles identify outliers. These data do not include the erosional effects of *Acanthaster solaris*.
