# Supplementary material for: Carbonate production of Micronesian reefs suppressed by thermal anomalies and Acanthaster as sea-level rises
Source: PLoS One. 2019 Nov 15;14(11):e0224887. doi: 10.1371/journal.pone.0224887 (PMC6857905; doi:10.1371/journal.pone.0224887)
Supplement: Attachment — Submitted filename: Review of van Woesik and Cacciapaglia.pdf [file pone.0224887.s003.pdf]

Review of van Woesik and Cacciapaglia

This manuscript provides carbonate budgets for two coral reefs in Micronesia, a region that is likely to be highly impacted by future sea-level rise. The manuscript is well-written, the methods are sound, and the results provide much needed data from an understudied region. Most of my comments are relatively minor, but I do have a couple of more substantial points, outlined in the following paragraphs, that I would like to see the authors address before publication.

Lines 114-117: While I don't disagree with the statement that sediment influx may negatively impact carbonate production at high levels though coral loss, it's not clear to me whether changing the sign of the sedimentation rate accurately deals with this issue. Sediment influx itself, as long as that sediment is retained, necessarily always contributes positively to accretion (see Montaggioni 2009, cited in this manuscript and Perry et al. 2013, Coral Reefs). The effect of smothering is coral mortality which should be reflected in the calcification component of the equation by reducing coral cover, unless the effect of sedimentation is to also cause sub-lethal effects on coral growth. I see that this calculation was published in a previous study, but I think a little more justification here would be helpful.

Lines 327-338: The estimated rates of reef accretion for the different islands and reef habitats really needs to be presented in a Table or a Figure, given that the comparison of these rates to RCP pathways is one of the main aims of the paper. I would suggest another Figure like Fig. 1 that plots accretion rates versus predicted rates of sea-level rise.

Line 271: Many of the inner reefs had a higher threshold, but there was also a higher range than in the outer reefs. This is worth noting, I think. Additionally, it looks like the only statistically significant difference was between the outer reefs and the patch reefs at Pohnpei. I would be clear about this. And relatedly,

Line 340: I would really like to see some direct discussion of *why* there is a higher threshold (and a higher range in that threshold) of coral cover in the inner reefs versus the outer reefs? There is some discussion later on (Lines 357-361) about what species are driving this difference, but it would be good to add a couple of sentences about the life-history characteristics of these species composition that driving this difference. What else is known about these species and their likely resilience to future disturbances? What implications does this have for our understanding of the ability of reefs elsewhere to keep pace with SLR? It would also be good to include a brief discussion of how the thresholds of coral cover for these islands compare with what has been found in studies elsewhere and why there are differences.

<u>Minor comments</u>:

Lines 33-34: compared with reefs in Pohnpei? Or reefs without *Acanthaster* on Kosrae?

Line 34: I would specify that this is for both Pohnpei and Kosrae.

Line 47: It would be nice to have a Figure, perhaps in the Supporting Information or as an inset for Figs. 2 and 3, that orients readers not familiar with Micronesia where these islands are located both in the broader Pacific and relative to each other.

Line 58: I would strongly suggest changing "accretion" to carbonate-production here and throughout this section. The values being reported here are carbonate production rates, which are not strictly comparable to reef accretion rates because accretion incorporates long-term compaction/erosion as well as voids that form within the reef. Estimates of modern "accretion" typically require knowledge about geomorphological factors such as the proportion of void space in the reef (see Perry et al. 2013); however, the relationship between reef accretion and carbonate production used in this paper seems reasonable as well.

Line 59: This is a *gross* carbonate production value, which is not comparable to the *net* production values discussed in the previous and following sentences.

Line 73: Need a space between *A.* and *solaris*

Line 115: I'd put this calcification rate in kg/m2/d for consistency

Lines 121-124: What data were the growth rates and densities of the corals based on? Are these data species-specific or generic-level? Can you provide a reference to this information?

Line 142: Why wasn't endolithic microbioerosion included (i.e., a standard value as in the Caribbean Reef Budget protocol or Perry and Morgan 2016)?

Line 164: What is the estimate of 9 hours per day of reeftime based on?

Line 189: Something is wrong with the wording of this sentence (the influence…has not been previously attempted). Perhaps change to something like: Studies aimed at quantifying the influence…have not been previously attempted.

Line 217: This should say something like: To convert *net carbonate production*, from equation 1, to vertical reef accretion…

Lines 237-238: It's worth noting I think that these differences were still within error of one another.

Line 244: Please make it clear here and for all of the figures that show net carbonate production that those data do not include the influence of *Acanthaster*, as was done in the Supplemental Figure captions.

Line 254: The difference between Net and Gross production was not significant, correct?

Line 256: It would be helpful to have this location labeled on the map in Fig. 3

Line 273: This is gross, taxon-specific, carbonate production, correct? I think it would be helpful if the y-axes of all of the figures indicated whether they are showing net or gross values.

Line 267: change "all" to "gross"

Line 332: This statement needs to be qualified. 11-12mm/yr is higher than average Holocene accretion rates in many (maybe most) locations, but there are many places in the Indo-Pacific where rates this high have been recorded, included in the reference cited here.

Lines 334-336: Please indicate what proportion of the reefs would (or wouldn’t) keep up with this scenario, rather than just the majority.

Line 371: which reduced coral cover overall? Of particular species?
