# Supplementary material for: Carbonate production of Micronesian reefs suppressed by thermal anomalies and Acanthaster as sea-level rises
Source: PLoS One. 2019 Nov 15;14(11):e0224887. doi: 10.1371/journal.pone.0224887 (PMC6857905; doi:10.1371/journal.pone.0224887)
Supplement: Attachment — Submitted filename: Reviewer response letter RvW & CC Sept 9 2019 Final.docx [file pone.0224887.s004.docx]

Dear Editor,

Thank you for sending our manuscript (PONE-D-19-15515 Carbonate production of Micronesian reefs suppressed by thermal anomalies and *Acanthaster* as sea-level rises) to the two expert reviewers. Their thoughts and comments have greatly improved the manuscript. Below we address each comment (in italics), with our response in normal font.

**Academic Editor**

We note that Figures 2, 3, 6 and 7 in your submission contain [map/satellite] images which may be copyrighted. If applicable, please specify in the figure caption text when a figure is similar but not identical to the original image and is therefore for illustrative purposes only.

**Response: We have changed the four figures so that they no longer show copyrighted images.**

**Reviewer #1**

This is an overall well-written and well-executed study documenting the net carbonate production budgets for islands in Micronesia. Most significantly, the authors have pioneered spatial kriging within an excellent random, stratified plot design to determine net carbonate budgets around the perimeters of the entire islands. I believe this aspect of the study is an exceptional contribution to the field and applaud the authors for this contribution in using these methods to determine island scale carbonate production budgets. However, I have some concerns with how sedimentation and Acanthaster erosion were quantified and the language surrounding the capacity for reef accretion to keep up with sea level rise. I think this is nonetheless and important study and contribution to the field and hope the authors find the below comments helpful for improving the submitted manuscript prior to publication.

1. Sedimentation##################################################################

   Lines 115–117/132–138: If sedimentation is high, how does that generate a negative term for the carbonate production equation? There’s more carbonate being dumped on the site so this should be positive… I see the clarification for this based on the influences of terrestrial sedimentation. However, if solid particles are settling on a reef then it’s adding material that will increase accretion. I agree that sedimentation could reduce calcification rates by some factor and organics could increase CaCO3 dissolution rates; however, I disagree with utilizing a baseline –0.4 kg CaCO3/m2/y as an appropriate adjustment for such affects considering that there is a material flux onto the reef. I think the sedimentation section should more accurately reflect what was actually measured in this study and the authors are welcome speculate that sedimentation decreased calcification rates nearshore if they wish to include such a conclusion in the manuscript but I do not think they should directly quantify the –0.4 kg CaCO3/m2/y in their budgets owing to sedimentation being present.

**Response 1: We agree with the reviewer; sedimentation does contribute to reef accretion, however we distinguish between low-level calcareous sedimentation derived from reef organisms and fine terrestrial-derived sedimentation that is known to smother corals. Our model incorporates the addition of low sedimentation as being a positive effect of reef growth, as the reviewer suggests, however we also remind readers of the threshold (> 5 mg cm^-2^ d^-1^) introduced by Rogers (1983) for terrestrial (siliciclastic) sedimentation that can compromise corals, and in turn reducing potential reef accretion. We only insert the reduction of -0.4 CaCO3 m^2^ y^-1^ where we recorded siliciclastic sedimentation, for example at Utwe in Kosrae, and not across all sites as the reviewer suggests. We have clarified this point in the methods and added the following reference:** **Rogers CS 1983 Sublethal and lethal effects of sediments applied to common Caribbean reef corals in the field *Mar. Pollut. Bull. 14: 378-382***

2. Acanthaster####################################################################

   Lines 189-217: I appreciate the author's efforts to include Acanthaster erorion in this study however this section neglects quite a bit of the literature on the effects of Acanthaster on coral reef growth and should therefore be restructured. For example, I see no evidence in the present manuscript that Acanthaster actually consumes the coral calcium carbonate. The authors should note that Eakin (1996) found that Acanthaster digests tissue without destroying skeletons and Glynn (1973) previously assessed densities of Acanthaster that would overwhelm the ability for corals to persist. Lastly, Henderson and Walbran (1992) found that Acanthaster CaCO3 skeletal fragments were preserved in reef sediments and therefore added to reef CaCO3 production. I recognize that there is an influence of reducing coral cover owing to consumption of corals by Acanthaster that would drive decreases in CaCO3 production indirectly through the reduction in corals sensu Glynn (1973); however, the equations presented in this section make it seem as though Acanthaster is actually consuming CaCO3 in an erosional sense- a finding that the aforementioned papers would contradict and the present study does not provide sufficient quantifiable evidence otherwise. If the goal is to estimate the loss in CaCO3 production owing to Acanthaster- why not estimate a mean loss rate of corals per annual as per your equations and apply this loss in coral cover to the coral carbonate production equation to estimate the lost capacity for producing calcium carbonate by corals? However, if this is the study design then there should be some consideration of coral recruitment and whether or not the coral cover is in a quasi-steady state through time.

Glynn, P. W. (1973). Acanthaster: effect on coral reef growth in Panama. Science, 180(4085), 504-506.

Eakin, C. M. (1996). Where have all the carbonates gone? A model comparison of calcium carbonate budgets before and after the 1982–1983 El Nino at Uva Island in the eastern Pacific. Coral Reefs, 15(2), 109-119.

Henderson, R. A., & Walbran, P. D. (1992). Interpretation of the fossil record of Acanthaster planci from the Great Barrier Reef: a reply to criticism. Coral Reefs, 11(2), 95-101.

**Response 2:** **The reviewer suggests that we “estimate a mean loss rate of corals per annual as per your equations and apply this loss in coral cover to the coral carbonate production equation to estimate the lost capacity for producing calcium carbonate by corals”. This is exactly what we did. We did not assume that *Acanthaster* erode reefs, because *Acanthaster* eat the soft-polyp tissue of corals. We estimated the mean loss rate of coral cover and subtracted that loss from gross production rather than from net production. The previous assessment by Glynn (1973) showed the density of *Acanthaster* that would overwhelm corals ability to persist, which is a very important metric and we have added the reference. However, we were interested at finding the density of *Acanthaster* at which the net accretion of a reef becomes negative, a metric relatable to the growing concerns of sea-level rise. We have clarified this point further in the methods.**

3. Accretion######################################################################

   Line 58 (and throughout): Accretion generally refers to vertical accretion (mm/y) whereas net carbonate production generally refers to (kg CaCO3/m^2/y). Because Perry et al (2013) calculates both accretion and net carbonate production rates, I think “accretion” should be switched to “net carbonate production” to be more consistent with the numbers from Perry et al (2013). It would be good to be consistent throughout the manuscript with respect to the use of accretion vs. net carbonate production terms.
   **Response 3:** **We have corrected the wording to be consistent throughout the document.**
4. Line 219: What is the value for alpha in this study? I visited the previous van Woesik and Cacciapaglia (2018) publication and the alpha term derived therein referred me to the supplementary data of that manuscript where it’s a single value of -0.01949. Is this the value used in the present study? If so please just put this value in the text in place of requiring a reader to jump through so many hoops to determine a single number.

**Response 4:** **Yes, alpha is still -0.****01949. We have included this value in the revised document, and it is in the code, which will be readily accessible to readers.**

5. Additionally, how does this calculation compare to the previously used values derived by Kinsey (1985) assuming a density of 2.9 g cm^-3 for calcium carbonate and average porosity of 50% and leveraged by Perry et al (2018) where they used 30-70% porosity with 50% reincorporation of parrotfish derived sediment back into the reef framework?

Kinsey (1985) Metabolism, calcification and carbon production. Proceedings of the fifth international coral reef congress, Tahiti, Vol 4.

Perry, C. T., Alvarez-Filip, L., Graham, N. A., Mumby, P. J., Wilson, S. K., Kench, P. S., ... & Januchowski-Hartley, F. (2018). Loss of coral reef growth capacity to track future increases in sea level. Nature, 558(7710), 396.

**Response 5: The estimates of the present study and Kinsey’s study are similar. For example, with a G of 10, 4, and 0.5, Kinsey (1985) estimated vertical growth at 6.9, 3, and 0.35 mm year (note that Kinsey used an average density of 2.9 g cm^-3^ and assumed a porosity of 50%, calculated as 2.9*.5=1.45, and then 10/1.45=6.9 mm year). Our estimates were 8, 3.6, and 0.49 mm year for G of 10, 4, and 0.5. However, notably, we measured the density of each coral species separately without estimating their porosity. On average the coral density is only approximately 2.1 g cm^-3^, and we therefore consider our estimates more accurate.**

6. Lines 331-345: Perhaps I am confused about how the authors dealt with sedimentation in this study, but if the net CaCO3 sediment transport to the inner reefs (and microbioerosion/CaCO3 dissolution) was not considered than in my opinion these statements are likely too strongly stated. I caution making strong statements on such matters when the accretion rates of the inner reefs weren’t directly quantified in this study (e.g., as in Yates et al 2017) and the budgets presented here do not appear to fully cover all of the terms necessary to quantify net carbonate accumulation (i.e., see Kleypas et al [2001]). Since accretion is estimated from the budgets and the relationship between net carbonate production budgets and accretion rate was based on just a few points, there is quite a bit of uncertainty in this value that should temper the conclusions drawn from it especially considering that such statements potentially impact human population centers associated with these reefs in Micronesia and set the precedent for the same standard elsewhere.

Perry, C. T., Kench, P. S., Smithers, S. G., Riegl, B. R., Gulliver, P., & Daniells, J. J. (2017). Terrigenous sediment-dominated reef platform infilling: an unexpected precursor to reef island formation and a test of the reef platform size–island age model in the Pacific. Coral Reefs, 36(3), 1013-1021.

Yates, K. K., Zawada, D. G., Smiley, N. A., & Tiling-Range, G. (2017). Divergence of seafloor elevation and sea level rise in coral reef ecosystems. Biogeosciences, 14(6), 1739.

Perry, C. T., Morgan, K. M., & Yarlett, R. T. (2017). Reef habitat type and spatial extent as interacting controls on platform-scale carbonate budgets. Frontiers in Marine Science, 4, 185.

Kleypas, J. A., Buddemeier, R. W., & Gattuso, J. P. (2001). The future of coral reefs in an age of global change. International Journal of Earth Sciences, 90(2), 426-437.

Eyre, B. D., Andersson, A. J., & Cyronak, T. (2014). Benthic coral reef calcium carbonate dissolution in an acidifying ocean. Nature Climate Change, 4(11), 969.

**Response 6: We have adjusted the statement (lines 331-334) to include the uncertainty of our study. See also Response 1 on how we approached sedimentation.**

7. Coral bleaching and reef-scale calcification##########################################

   Line 375: There is also a growing literature on coral reef net ecosystem calcification and coral bleaching that provide critical information to the dynamics of carbonate production and thermal stress events and could be cited here. Please see the following papers:

   Kayanne, H., Hata, H., Kudo, S., Yamano, H., Watanabe, A., Ikeda, Y., ... & Saito, H. (2005). Seasonal and bleaching‐induced changes in coral reef metabolism and CO2 flux. Global Biogeochemical Cycles, 19(3).

   Watanabe, A., Kayanne, H., Hata, H., Kudo, S., Nozaki, K., Kato, K., ... & Yamano, H. (2006). Analysis of the seawater CO2 system in the barrier reef‐lagoon system of Palau using total alkalinity‐dissolved inorganic carbon diagrams. Limnology and Oceanography, 51(4), 1614-1628.

   DeCarlo, T. M., Cohen, A. L., Wong, G. T., Shiah, F. K., Lentz, S. J., Davis, K. A., ... & Lohmann, P. (2017). Community production modulates coral reef pH and the sensitivity of ecosystem calcification to ocean acidification. Journal of Geophysical Research: Oceans, 122(1), 745-761.

   Courtney, T. A., De Carlo, E. H., Page, H. N., Bahr, K. D., Barro, A., Howins, N., ... & Andersson, A. J. (2018). Recovery of reef‐scale calcification following a bleaching event in Kāne'ohe Bay, Hawai'i. Limnology and Oceanography Letters, 3(1), 1-9.

   McMahon, A., Santos, I. R., Schulz, K. G., Scott, A., Silverman, J., Davis, K. L., & Maher, D. T. (2019). Coral reef calcification and production after the 2016 bleaching event at Lizard Island, Great Barrier Reef. Journal of Geophysical Research: Oceans.

**Response 7: We thank the reviewer for the suggested literature; we have read these papers and have included three of the most relevant citations in the revised manuscript.**

Minor comments################################################################

8. Lines 88–95: excellent site selection design!!!
   **Response 8: Thank you**
9. Lines 129-130: Isn’t the gross carbonate production in g/cm^2/y and the resulting units after conversion kg/m^2/y?

**Response 9: We have added the temporal component to the converted unit.**

10. Please maintain consistency with units as “y” and “yr” are used interchangeably throughout the manuscript

**Response 10: We have changed the unit to yr throughout the text.**

11. The erosion section and equations therein are quite similar to Perry et al. (2012) and should be cited as such where appropriate.

**Response 11: The parts of this section that were derived from Perry et al. (2012) have been cited as such in the revised text.**

12. Line 199: Shouldn’t the kg/m^2 be per year?

**Response 12: We have corrected this issue.**

13. Line 256: Couldn’t this just in part be driven by the somewhat arbitrarily assigned -0.4 kg/m2/y rate applied to the high sedimentation areas?

**Response 13. Yes, absolutely, that is what we were trying to imply. We did not measure the exact levels in sedimentation in this study, although we did record terrestrial sedimentation in some sites in Kosrae. Based on these data and on previous work on the negative effects of terrestrial discharge on coral reefs, we assigned the -0.4 value to convey the point that discharge from cleared forests reduces carbonate production on reefs. We have clarified this issue in the revised manuscript.**

14. Alternatively, how did the authors account for the very large expanses of sand that typically characterize inshore lagoonal environments?
    **Response 14. We did not come across sites that were primarily sand on the nearshore sites of Pohnpei and Kosrae. If we had, which was part of the protocol, we would have reverted to the closest reef site.**
15. Line 311: add space to “reefs,and”

**Response 15. We have corrected the typo.**

16. Line 362-364: This is an excellent point and is a feature of geological perspectives of coral reefs. For example, please see Engels (2004) and Chapter 6 Montaggioni and Braithwaite (2009) for potential citations for this.

Engels, M. S., Fletcher III, C. H., Field, M. E., Storlazzi, C. D., Grossman, E. E., Rooney, J. J., ... & Glenn, C. (2004). Holocene reef accretion: southwest Molokai, Hawaii, USA. Journal of Sedimentary Research, 74(2), 255-269.

Montaggioni, L. F., & Braithwaite, C. J. (2009). Quaternary coral reef systems: history, development processes and controlling factors (Vol. 5). Elsevier.

**Response 16. Thank you, we have read this literature and added the citations.**

**Reviewer #2**

This manuscript provides carbonate budgets for two coral reefs in Micronesia, a region that is likely to be highly impacted by future sea-level rise. The manuscript is well-written, the methods are sound, and the results provide much needed data from an understudied region. Most of my comments are relatively minor, but I do have a couple of more substantial points, that I would like to see the authors address before publication. Both are outlined in the document attached.

17. Lines 114-117: While I don’t disagree with the statement that sediment influx may negatively

impact carbonate production at high levels though coral loss, it’s not clear to me whether

changing the sign of the sedimentation rate accurately deals with this issue. Sediment influx

itself, as long as that sediment is retained, necessarily always contributes positively to accretion

(see Montaggioni 2009, cited in this manuscript and Perry et al. 2013, Coral Reefs). The effect of

smothering is coral mortality which should be reflected in the calcification component of the

equation by reducing coral cover, unless the effect of sedimentation is to also cause sub-lethal

effects on coral growth. I see that this calculation was published in a previous study, but I think a

little more justification here would be helpful.

**Response 17. Please refer to Response 1 above where we addressed this comment in detail. Terrestrial sedimentation can cause sub-lethal effects and cause a reduction in the rate of net carbonate production and hence in reef accretion.**

18. Lines 327-338: The estimated rates of reef accretion for the different islands and reef habitats

really needs to be presented in a Table or a Figure, given that the comparison of these rates to

RCP pathways is one of the main aims of the paper. I would suggest another Figure like Fig. 1

that plots accretion rates versus predicted rates of sea-level rise.

**Response 18. We have added a new supplementary figure (Figure S7) comparing the vertical accretion of the habitats similar to figure 1b, with horizontal lines representing the rates of the various RCP scenarios.**

19. Line 271: Many of the inner reefs had a higher threshold, but there was also a higher range than

in the outer reefs. This is worth noting, I think. Additionally, it looks like the only statistically

significant difference was between the outer reefs and the patch reefs at Pohnpei. I would be

clear about this.

**Response 19. We clarified this issue in the revised text.**

20. Line 340: I would really like to see some direct discussion of why there is a higher threshold (and

a higher range in that threshold) of coral cover in the inner reefs versus the outer reefs? There is

some discussion later on (Lines 357-361) about what species are driving this difference, but it

would be good to add a couple of sentences about the life-history characteristics of these species

composition that driving this difference. What else is known about these species and their likely

resilience to future disturbances? What implications does this have for our understanding of the

ability of reefs elsewhere to keep pace with SLR? It would also be good to include a brief

discussion of how the thresholds of coral cover for these islands compare with what has been

found in studies elsewhere and why there are differences.

**Response 20. We have added discussion on the higher threshold and a greater uncertainty of those thresholds.**  **We do not wish, however, to speculate on the nature of different life-history strategies, because we see major problems with such an approach. Without going into a major critique of the use of simple life-history strategies, and the problems that these simple categories have created we prefer to report the coral species and genera.**

Minor comments:

21. Lines 33-34: compared with reefs in Pohnpei? Or reefs without Acanthaster on Kosrae?

**Response 21. We have clarified that the comparison is with Kosraen reefs without Acanthaster.**

22. Line 34: I would specify that this is for both Pohnpei and Kosrae.

**Response 22. We have clarified this point.**

23. Line 47: It would be nice to have a Figure, perhaps in the Supporting Information or as an inset

for Figs. 2 and 3, that orients readers not familiar with Micronesia where these islands are

located both in the broader Pacific and relative to each other.

**Response 23. We have created a map with an inset for the islands as Figure S1.**

24. Line 58: I would strongly suggest changing “accretion” to carbonate-production here and

throughout this section. The values being reported here are carbonate production rates, which are

not strictly comparable to reef accretion rates because accretion incorporates long-term

compaction/erosion as well as voids that form within the reef. Estimates of modern “accretion”

typically require knowledge about geomorphological factors such as the proportion of void space

in the reef (see Perry et al. 2013); however, the relationship between reef accretion and carbonate

production used in this paper seems reasonable as well.

**Response 24.** **We have adjusted these statements to maintain consistency throughout the paper, using the term carbonate production.**

25. Line 59: This is a gross carbonate production value, which is not comparable to the net

production values discussed in the previous and following sentences.

**Response 25.** **We have changed this to the net carbonate production.**

26. Line 73: Need a space between A. and solaris

**Response 26. The typo has been corrected.**

27. Line 115: I’d put this calcification rate in kg/m2/d for consistency

**Response 27. We have corrected the calcification rate for consistency.**

28. Lines 121-124: What data were the growth rates and densities of the corals based on? Are these

data species-specific or generic-level? Can you provide a reference to this information?

**Response 28. The growth rates, densities, and morphological adjustments were all following van Woesik and Cacciapaglia 2018. The cross-referencing has been clarified.**

29. Line 142: Why wasn’t endolithic microbioerosion included (i.e., a standard value as in the

Caribbean Reef Budget protocol or Perry and Morgan 2016)?

**Response 29. We did not measure endolithic microbioerosion, but know from the literature (including, Journal of Experimental Marine Biology and Ecology 252 (2000) 1–14) that rates of bioerosion and micro-erosion vary spatially. Therefore, without that information we did not think that applying a standard value across all sites was useful.**

30. Line 164: What is the estimate of 9 hours per day of reeftime based on?

**Response 30. This was based on previous estimates by Mumby and coworkers who suggested that the grazing hours of parrotfishes were on average approximately nine hours. We have clarified this point in the revised manuscript.**

31. Line 189: Something is wrong with the wording of this sentence (the influence...has not been

previously attempted). Perhaps change to something like: Studies aimed at quantifying the

influence...have not been previously attempted.

**Response 31. We have re-arranged the sentence.**

32. Line 217: This should say something like: To convert net carbonate production, from equation 1,

to vertical reef accretion...

**Response 32. We have rewritten the sentence for clarity.**

33. Lines 237-238: It’s worth noting I think that these differences were still within error of one

another.

**Response 33. We agree and have addressed this issue in Response 20.**

34. Line 244: Please make it clear here and for all of the figures that show net carbonate production

that those data do not include the influence of Acanthaster, as was done in the Supplemental

Figure captions.

**Response 34. We have clarified the captions.**

35. Line 254: The difference between Net and Gross production was not significant, correct?

**Response 35. The difference between gross production and net carbonate production at Utwe bay was significantly different (p<0.001) than the average difference between gross and net production at all sites.**

36. Line 256: It would be helpful to have this location labeled on the map in Fig. 3

**Response 36. We have added a map inset of the study sites to the supplementary document based on suggestions from Reviewer 1.**

37. Line 273: This is gross, taxon-specific, carbonate production, correct? I think it would be helpful

if the y-axes of all of the figures indicated whether they are showing net or gross values.

**Response 37. We have made the axes reflect net or gross carbonate production accordingly.**

38. Line 267: change “all” to “gross”

**Response 38. This has been corrected.**

39. Line 332: This statement needs to be qualified. 11-12mm/yr is higher than average Holocene

accretion rates in many (maybe most) locations, but there are many places in the Indo-Pacific

where rates this high have been recorded, included in the reference cited here.

**Response 39. Our statement has now been qualified in the revised manuscript.**

40. Lines 334-336: Please indicate what proportion of the reefs would (or wouldn’t) keep up with

this scenario, rather than just the majority.

**Response 40. We have changed the wording to emphasize that the average outer reef can barely keep up with sea-level rise, and have added a supplementary figure, which includes the likelihood, with uncertainty, of reefs in each habitat keeping up with sea-level rise under the four Representative Concentration Pathway scenarios (i.e., 2.6, 4.5, 6.0, and 8.5 W m^-2^).**

41. Line 371: which reduced coral cover overall? Of particular species?

**Response 41. We have clarified this statement in the revised manuscript.”** The lower carbonate production rates are most likely a result of reduced *Acropora* cover caused by the recent thermal-stress events on both Kosrae and Pohnpei in 2016 and 2017”.

**Our thanks extend to the editor and both reviewers for pointing out ambiguities and other issues that we have clarified in the revised manuscript.**

**Robert van Woesik and Chris Cacciapaglia**
